# Stigma as a Barrier to Mental Health Service Use Among Female Sex Workers in Switzerland

**DOI:** 10.3389/fpsyt.2019.00032

**Published:** 2019-02-05

**Authors:** Mara Zehnder, Jochen Mutschler, Wulf Rössler, Michael Rufer, Nicolas Rüsch

**Affiliations:** ^1^Department of Psychiatry, Psychotherapy and Psychosomatics, Psychiatric University Hospital, University of Zürich, Zurich, Switzerland; ^2^Privatklinik Meiringen, Meiringen, Switzerland; ^3^Department of Psychiatry and Psychotherapy, Charité Universitätsmedizin, Berlin, Germany; ^4^Laboratory of Neuroscience, Institute of Psychiatry, Universidade de São Paulo, São Paulo, Brazil; ^5^Department of Psychiatry II, University of Ulm and BKH Günzburg, Ulm, Germany

**Keywords:** sex work, prostitution, stigma, barriers to care, service use, mental health, perceived need for treatment

## Abstract

**Background:** Many sex workers suffer from mental health problems, but do not seek help.

**Aim:** To examine stigma-related and non stigma-related barriers to care and perceived need for treatment among female sex workers in Switzerland.

**Methods:** Mental health service use, barriers to care, perceived need and presence of illness, symptoms, and psychiatric diagnoses were assessed among 60 female sex workers in Zürich, Switzerland.

**Outcomes:** Mental health service use was defined as use of psychiatric medication, psychotherapy, or substance use services for at least 1 month during the past 6 months.

**Results:** Adjusting for symptom levels, mental health service use was predicted by lower stigma-related, not by structural, barriers as well as by more perceived need for treatment and higher age.

**Clinical Implications:** Sex workers with mental health problems would benefit from non-stigmatizing mental health care as well as from interventions to reduce public and self-stigma associated with mental illness and sex work.

**Strengths and Limitations:** Limitations are the cross-sectional data, limited sample size, and recruitment from an information center for sex workers.

**Conclusion:** Interventions that aim to increase mental health service use among sex workers should take stigma variables into account.

## Introduction

Mental health problems are common among sex workers (1, 2). In a previous Swiss study one half of female sex workers reported at least one mental disorder in the past year (3), which is higher than the 12-month prevalence of about one third for any mental disorder in the general population (4). In the Swiss general population only about 7% of all women receive mental health care (5). Rates of mental health service use among female sex workers in Switzerland are unknown, but international data suggest that unmet health care needs are higher among sex workers than in the general population (6).

Many sex workers have a double stigmatized identity due to their work and common mental health problems (7). Despite high rates of unmet mental healthcare needs (6) specific interventions for sex workers with mental health problems are lacking. Previous research with sex workers suggested that instrumental barriers, e.g., lack of time or money, as well as stigma-related variables (fear of being labeled or shame) were a barrier to mental health service use (6, 7). Studies among people with mental illness outside the sex industry highlighted perceived need for treatment as a determinant of help-seeking (8).

Previous research on predictors of mental health service use among sex workers is inconclusive so far, and this especially applies to Europe. We therefore conducted a study among female sex workers in Switzerland to examine predictors of mental health service use. We expected that higher perceived need for treatment and lower stigma-related as well as non stigma-related barriers to care would predict help-seeking, adjusted for symptom levels.

## Methods

### Participants

Sixty female sex workers were recruited in a church-funded information center for sex workers in the inner-city red light district of Zürich, Switzerland. The center offers advice on health and prevention, legal issues, as well as assistance in dealing with paperwork and government agencies. German language courses and free lunch are also provided. There is no system of regular referrals of clients to mental health services. The study was approved by the local ethics committee (Kantonale Ethikkomission Zürich). All participants provided written informed consent and were reimbursed for their time during assessments. Participants were female and 18 years or above (*M* = 38.4 years, *SD* = 11.7). Most participants (*n* = 46, 78%) came from Hungary, Nigeria or Latin America. Work settings varied, including streets, bars and brothels.

### Measures

All questionnaire data were collected during one-to-one interviews. Barriers to care were assessed using the 30-item Barriers to Access to Care Evaluation scale [BACE 9], rating barriers to professional care in the past 6 months from 0/not at all to 3/a lot. The BACE contains a 12-item stigma-related (*M* = 1.1; *SD* = 0.9; Cronbach's alpha 0.92; e.g., “feeling embarrassed or ashamed”) and an 18-item non-stigma-related subscale (structural and attitudinal barriers, *M* = 1.1, *SD* = 0.4; Cronbach's alpha 0.65; e.g., “thinking appointments take too much time or are inconvenient”). Perceived need for treatment and perceived presence of illness were measured with the 6-item Perceived Need for Treatment subscale and the 4-item Presence of Illness subscale of the Self-Appraisal of Illness Questionnaire (10), with higher mean scores from 1 to 4 indicating more perceived need (*M* = 2.4, *SD* = 0.8; alpha 0.87) or a perceived stronger presence of illness, respectively (*M* = 2.0, *SD* = 0.8; alpha 0.96).

Using a previously validated self-report measure of mental health service use (11), participants indicated whether or not, and for how long, they had used mental health care in the past 6 months in terms of psychiatric medication, psychotherapy/counseling, or substance use services. There was a bimodal distribution, i.e., most participants had used the respective service continuously over 6 months or not at all. For a logistic regression, we therefore created one dependent binary variable (using one or more of these three services not at all/only once, vs. continuously for 1–6 months). Psychopathology was assessed using a 9-item version of the Symptom Check List–Revised (12), and axis-I mental disorders with the Mini International Neuropsychiatric Interview (13).

## Results

Among all 60 participants, 20 (33%) had used mental health care continuously for at least 1 month during the past 6 months, while 40 (67%) had not. Out of those 45 with at least one psychiatric diagnosis, 20 (44% of 45) had used mental health care and 25 (56% of 45) had not. Forty-five (75%) participants had at least one mental disorder, most commonly affective or anxiety disorders. The most common diagnoses were major depression (*n* = 34, 54%), dysthymia (*n* = 25, 42%), and panic disorder (*n* = 22, 37%).

In a multiple logistic regression and adjusted for symptoms, mental health service use was predicted by a lower level of stigma-related, not of non-stigma related, barriers to care, by more perceived need for treatment, higher perceived presence of illness, and by higher age (Table 1; Nagelkerke's *R*^2^ = 0.73). The non-significant Hosmer-Lemeshow-Test (χ^2^ = 2.85, *p* = 0.94) indicates adequate model fit. The regression model correctly predicted 87% of those who did not, and 85% of those who did, use mental health services. When repeating the regression among only those 45 participants with at least one mental disorder, results remained largely unchanged (stigma-related barriers, *p* = 0.03; perceived need for treatment, *p* = 0.009; presence of illness, *p* = 0.07; symptoms, *p* = 0.09; age, *p* = 0.02).

**Table 1:** Logistic regression on mental health service use (i.e., use of psychopharmacotherapy or psychotherapy or substance use services in the past 6 months, for at least 1 month; *N* = 60).

**Independent variables**	**B (SE)**	**Odds ratio**	***p***
Stigma-related barriers to care (BACE)	−2.38 (1.09)	0.09	0.03
Non stigma-related barriers to care (BACE)	1.47 (1.48)	4.34	0.32
Perceived need for treatment (SAIQ)	3.16 (1.08)	23.6	0.004
Perceived presence of illness (SAIQ)	2.78 (1.36)	16.1	0.04
Symptoms (SCL-9)	1.01 (0.74)	2.74	0.17
Age	0.15 (0.07)	1.16	0.02

## Discussion

The prevalence of common mental disorders among female sex workers in our study was high, confirming a previous Swiss study (3), and elevated compared to the general population. The majority of participants, even of those with a manifest mental disorder, had not sought mental health care in the past 6 months. This can also be said about the Swiss general population (5), but it highlights the sizeable treatment gap in this particularly vulnerable population.

Our findings underline the role of stigma-related barriers to care while previous research focused on structural barriers, such as lack of time or financial resources (6); the latter were not associated with service use in our study. Interventions to increase mental health service use of female sex workers could therefore address stigma as a barrier to treatment. This could involve setting up non-stigmatizing mental health care environments as well as interventions to reduce self-stigma and shame. These interventions could also try to increase mental health literacy as a means to increase perceived need for treatment and knowledge about the benefits of available treatments (14).

Several limitations of our study need to be considered. Recruitment among sex workers, a fluctuating and hard-to-reach population, is notoriously difficult; due to recruitment from an information center our sample is unlikely to be representative and thus findings cannot be generalized. The cross-sectional data preclude conclusions on causality. Barriers to care were assessed by a generic measure, and information for barriers to care specific for sex workers was not collected. Finally, due to our limited sample size odds ratios in the regression model may be overestimated.

Despite these limitations, our study suggests that stigma and the subjective appraisal of illness are relevant barriers to care seeking. Interventions that aim to increase mental health service use among the vulnerable population of sex workers with mental health problems should target these variables.

## Data Availability Statement

Study participants provided written informed consent on the condition that their data would be available only to our research group. Therefore, data cannot be made publicly available.

## Author Contributions

MZ, JM, WR, and NR contributed to study design. MZ led recruitment and data collection, supported by JM and NR. MZ and NR analyzed the data. NR wrote the first draft of the manuscript. All authors contributed to the manuscript's content and read and approved the submitted version.

### Conflict of Interest Statement

The authors declare that the research was conducted in the absence of any commercial or financial relationships that could be construed as a potential conflict of interest.
